# Comparing different environmental enrichments for improving the welfare and walking ability of male turkeys

**DOI:** 10.1371/journal.pone.0285347

**Published:** 2023-05-09

**Authors:** Yiru Dong, Gregory S. Fraley, Janice M. Siegford, Fengqing Zhu, Marisa A. Erasmus

**Affiliations:** 1 Department of Animal Sciences, Purdue University, West Lafayette, IN, United States of America; 2 Department of Animal Science, Michigan State University, East Lansing, MI, United States of America; 3 Elmore Family School of Electrical and Computer Engineering, Purdue University, West Lafayette, IN, United States of America; University of Life Sciences in Lublin, POLAND

## Abstract

This study investigated age-related changes in turkey welfare measures (wounds, feather quality (**FQ**), feather cleanliness, and footpad condition (**FCON**)) and walking ability (gait) as influenced by different types of environmental enrichment (**EE**). Tom turkeys (n = 420) were randomly assigned to: straw bale (**S**), platform (**P**), platform + straw bale (**PS**), pecking block (**B**), tunnel (**T**) or control (**C**; no enrichment) group. Welfare measures and gait were assessed at 8, 12, 16 and 19 wk and analyzed using PROC LOGISTIC with Firth bias-correction. Better wing FQ with age was observed in turkeys in S and T groups. Turkeys in the S group had better wing FQ at 16 (P = 0.028) and 19 wk (P = 0.011) vs. 8 wk. Wing FQ (P = 0.008) was better at 19 vs. 8 wk for T turkeys. FCON worsened over time for turkeys in all treatment groups except for the S group. FCON was worse at 19 vs.8 wk for P (P = 0.024), PS (P = 0.039), B (P = 0.011), T (P = 0.004) and C (P = 0.014) turkeys and was worse at 19 vs. 12 wk for B (P = 0.038), T (P = 0.015) and C (P = 0.045) turkeys. FCON was worse at 19 vs. 16 wk for T (P = 0.007) and C (P = 0.048) turkeys. FCON was also worse at 16 vs. 8 wk for B (P = 0.046) turkeys. Gait worsened with increasing age in all treatment groups. Gait was worse at 19 wk for S (P < 0.001), P (P < 0.001), PS (P < 0.001) and B turkeys (P < 0.001) vs. earlier ages, while gait in T (P < 0.001) and C turkeys (P < 0.001) worsened starting at 16 wk.

## Introduction

Injurious pecking and leg health issues are among the major welfare and economic concerns of commercial turkey production. Injurious pecking among turkeys includes aggressive pecking (head pecking), feather pecking, and cannibalism. Aggressive pecking is particularly problematic in turkey toms and may lead to death in severe cases [1]. Severe feather pecking is a forceful pecking behavior that may involve removal and possible consumption of pulled feathers and leads to plumage damage and feather loss [1, 2]. Cannibalism can develop independently or following severe feather pecking if bleeding occurs at the site of feather pecking, which may result in tissue damage and even death of pecked birds [3]. In addition to welfare issues, injurious pecking can lead to economic losses, such as those resulting from body heat loss and the resulting increased feed intake to maintain thermoregulation, thereby leading to reduced feed efficiency. Injurious pecking can also result in carcass damage and downgrading, as well as increased culling and mortality, which reduce production efficiency [4–6].

Beak treatment is a common method of controlling injurious pecking in commercial turkey production [7]. Hot-blade trimming was standard practice in the past and can cause both acute and chronic pain. Infrared beak treatment is currently used to remove turkeys’ beak tissue without creating an open wound [7], and some studies have reported no evidence of pain or stress as indicated by beak-related behaviors and heterophil/lymphocyte ratios [8]. When examining feather damage associated with injurious pecking behavior, infrared beak treatment did not have a consistent effect on feather condition over a 12-wk period, with male sham-treated control birds having improved feather condition at 8 wk compared to beak-treated birds, while female infrared beak treated birds had better feather condition at 12 wk compared to controls [8]. Although it is common practice to treat turkeys’ beaks immediately after hatch to reduce the severity of injurious pecking, feather pecking continues to be problematic.

In addition to injurious pecking, impaired walking ability (gait) and lameness are other major welfare problems that are consistently listed among the top ten health concerns of commercial turkeys [9]. Affected turkeys can suffer from pain, difficulty in accessing resources such as feed and water, and reduced ability to escape from their conspecifics because of impaired locomotor ability [10], which in turn can lead to these turkeys becoming targets of injurious pecking. Turkeys with lameness issues may have lower body weights, resulting in economic losses [11]. Various factors contribute to gait impairment, such as rapid weight gain and breast muscle growth [12, 13], environment (e.g. temperature and humidity) and management conditions, slippery flooring, diet, and photoperiod [2, 12].

One possible method to reduce injurious pecking behavior and improve gait is to provide environmental enrichment. The use of environmental enrichment is becoming increasingly common as various animal welfare certification and assurance programs require the use of enrichment (e.g. Global Animal Partnership [14], American Humane Certified™ [15], Certified Humane® [16], and Farm Animal Care Training and Auditing [17]); however, science on the effectiveness of environmental enrichment for turkeys is lacking. Studies with turkeys have either examined only the effects of environmental enrichment on the prevalence of injurious pecking behavior and pecking injuries (e.g. [5, 18–20]), or examined only one type of environmental enrichment (e.g. [5, 21, 22]). To our knowledge, Sherwin et al. [21] is the only study that examined the effects of environmental enrichment on both pecking related injuries and musculoskeletal function of turkeys, but this study was conducted over 20 years ago using a strain of turkeys that differs in growth rate and productivity from modern commercial turkeys.

While previous research has provided some insights into the effectiveness of environmental enrichment to reduce injurious pecking in turkeys, several key questions remain: (1) what are the effects of different types of environmental enrichment on turkey gait; and (2) how do welfare and gait change with age when turkeys are provided with different types of environmental enrichment. Therefore, the objective of this study was to investigate age-related changes in turkey welfare measures and gait as influenced by five different types of environmental enrichment.

## Materials and methods

### Animals and housing

All procedures used in this study were approved by the Institutional Animal Care and Use Committee at Purdue University (PACUC 1904001883). A total of 420 infrared beak-trimmed tom turkeys (Nicolas Select, Aviagen Turkeys, Lewisburg, West Virginia, USA) were obtained from a commercial hatchery at 1 d of age and housed at the Purdue Animal Sciences Research and Education Center. From 1 to 11 d of age, the turkey poults were housed in 4 brooding rings with 105 birds per ring, and then randomly assigned to 24 littered (wood shavings) pens (measuring 3.05 m by 2.44 m) located in two rooms within the same barn with 16 to 18 birds per pen (stocking density 2.15 to 2.42 birds/m^2^). Each pen was supplied with a hanging feeder and two bell drinkers, providing standard commercial feed and water ad libitum. Lighting and temperature were maintained according to standard industry practice [23]. For the first 24 hours, birds were provided with 24 h of light. Thereafter, lighting was adjusted to a final photoperiod of 16 h light and 8 h of darkness by the fourth day. A minimum light intensity of 40 lux was provided. Room temperature was reduced weekly according to the Aviagen guidelines [23]; birds were initially brooded at 30°C, and thereafter the temperature was gradually decreased to a final temperature of 13°C at 14 weeks. Turkeys were kept in their assigned pens until 19 wk when they were processed at the Purdue University Butcher Block using electrical water bath stunning followed by exsanguination, similar to procedures used in commercial processing facilities. During the study, all efforts were made to alleviate suffering, and animals that appeared ill, injured or had gait impairments affecting their ability to reach food and water were evaluated and humanely euthanized using carbon dioxide or a non-penetrating captive bolt [24] if necessary.

### Environmental enrichment

**Fig 1:**
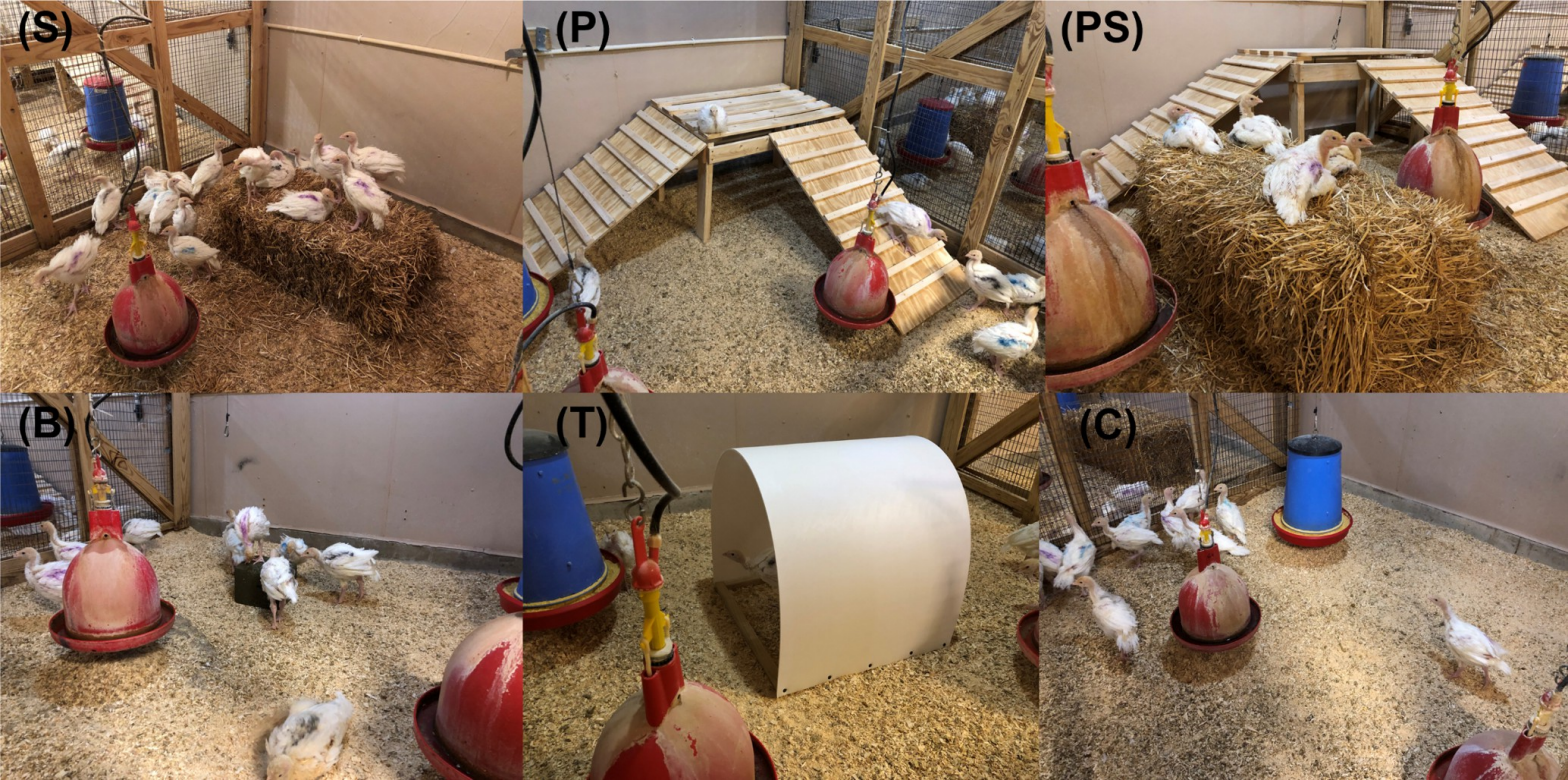
Depiction of the enrichment for each treatment group. S = straw bale, P = platform, PS = platform + straw bale, B = pecking block, T = tunnel, C = control.

Birds were randomly assigned to six treatment groups with 4 pens per treatment group, including five enrichment groups (straw bale, platform, platform + straw bale, pecking block and tunnel; Fig 1) and a control group. Environmental enrichments were provided starting at 4 wk.

Straw bale group (**S**): one straw bale (1.02 m length × 0.51 m width × 0.30 m height) was provided in the pen. Straw bales were replaced when the top of the bale collapsed and turkeys were no longer able to jump on the bale. Partially destroyed bales were assessed and replaced as necessary on a case-by-case basis. When replacing the bales, loose straw was also removed from the pen.

Platform group (**P**): one wooden platform with two ramps (overall size: 2.04 m length × 1.65 m width × 0.61 m height) was provided in the pen (platform size: 0.99 m length × 0.61 m width × 0.61 m height; ramp size: 1.22 m length × 0.61 m width, 30-degree angle relative to the ground). The ramps were at a 30-degree angle to the ground and had wooden strips (6 cm apart) on them to provide birds with traction for waking up the ramps.

Platform + straw bale group (**PS**): one wooden platform with two ramps (as described above) and one straw bale were provided in the pen. Straw bales were replaced using the same procedure as for the S group.

Pecking block group (**B**): one rectangular pecking block (0.23 m length × 0.23 m width × 0.18 m height) was provided in the pen (provided by Cargill Turkeys, AR, USA). New pecking blocks were placed into the pens when the old blocks were destroyed.

Tunnel group (**T**): one tunnel (0.61 m length x 0.61 m width x 0.58 m height) was provided in the pen. The tunnel was constructed of corrugated plastic sheets and a wood frame.

Control group (**C**): no enrichment was provided in the pen.

### Welfare assessment

Turkeys’ welfare status (wounds on head, neck, snood, back and tail; beak abnormalities; feather quality; feather cleanliness; and footpad condition) and body weight were assessed at 8, 12, 16 and 19 wk of age using a scoring system adapted from the AWIN turkey welfare assessment protocol [25], Welfare Quality® Assessment Protocol for Poultry [26] and G.A.P.’s 5-step® Animal Welfare Standards for Turkeys [15] (Table 1). At 8 and 19 wk, welfare status and body weight were assessed and recorded for all turkeys. At 12 and 16 wk, half of the birds from each pen were randomly selected and assessed for welfare status and body weight (210 birds in total at 12 wk and 205 birds in total at 16 wk). Feather cleanliness of the wings and tail were added to assessments starting at 16 wk. Welfare measures were scored on a scale of 0 to 1 (feather cleanliness) or 0 to 2 (beak abnormalities, wounds, feather quality, footpad condition) where 0 was the best or ideal condition and a 1 or 2 indicated a worsening condition for that specific characteristic. For welfare and gait assessments, observers attended an on-farm training for scoring turkeys and scored the same birds before each assessment until consensus was reached, after which they continued to score turkeys independently.

**Table 1:** Turkey welfare scores and definitions [14, 25–27].

Condition	Score	Definition
Wounds	0	Best: No wounds present
	1	Moderate: < 3 fresh pecks or scratches present
	2	Worst: ≥ 3 fresh pecks or scratches present
Beak abnormalities (uneven growth of the upper and lower mandibles, cross beak, (sharp or jagged mandible tip, permanent open beak, tubercular swelling formation)	0	Best: No beak trim, no abnormalities present
	1	Moderate: Beak trimmed, with mild abnormalities
	2	Worst: Beak trimmed, with severe abnormalities
Feather quality	0	Best: No or slight wear, (nearly) complete feathering (only single feathers lacking).
	1	Moderate: < 50% of the feathers are damaged (worn, deformed) or one or more featherless areas < 5 cm in diameter at the largest extent.
	2	Worst: At least one featherless area ≥ 5 cm in diameter at the largest extent; or ≥ 50% of the feathers are damaged (worn, deformed).
Feather cleanliness	0	Clean: Clean and unstained down or feathers, or light discoloration of feathers from dust
	1	Dirty: Very clear and dark staining of the back, wing and/or tail feathers, not including light discoloration of feathers from dust, covering at least 50% of the body area
Footpad condition	0	Best: No lesion, no discoloration, no scars. Only mild hyperkeratosis
	1	Moderate: mild and/or superficial lesions. Visible hyperkeratosis (thickened skin). Superficial erosions, papillae. Discoloration of the footpad.
	2	Worst: severe and/or deep lesions. Ulcers and scabs.

### Gait assessment

Turkeys’ gait was examined at 8, 12, 16 and 19 wk of age. Gait was scored for a minimum of six steps taken in the home pen. Gait scores were determined using the 6-point system (0–5) developed by Kestin et al. [28] and modified by Vermette et al. [29] and Garner et al. [30], where 0 represents no impairment in gait and 5 represents complete lameness. The detailed gait scoring system can be found in Table 2. After scoring the turkey’s gait, both legs of the turkey (feet and toes) were examined and any abnormalities were recorded (Table 3).

**Table 2:** Descriptions of gait scores [28–30].

GS	Degree of impairment	Description
0	None	Straight legs. Smooth, fluid locomotion. The foot is furled while raised.
1	Detectable, but unidentifiable abnormality	The bird is unsteady, or wobbles when it walks. However, the problem leg cannot be identified in the first 20 s of observation. The bird readily runs from the observer in the pen. The foot may remain flat when raised, but the rest of the stride is fluid and appears unimpaired. Gait appears unstable (shaky or stomping).
2	Identifiable abnormality that has little impact on overall function	The leg producing the gait defect can be identified within 20 s of observation. However, the defect seems to have only a minor impact on biological function. The bird will run from the observer spontaneously or if touched or nudged. If the bird does not run at full speed, it runs, walks, or remains standing for at least 15 s after the observer in the pen has ceased to move towards or nudge it. Birds in this and previous scores are often observed to scratch their face with their feet, indicating little impact on function. (The most common abnormality in this score is for the bird to make short, quick, unsteady steps with one leg, where the foot remains flat during the step).
3	Identifiable abnormality that impairs function	Although the bird will move away from the observer when approached, touched, or nudged, it will not run, and squats within 15 s or less of the observer in the pen ceasing to approach or nudge it.
4	Severe impairment of function, but still capable of walking	The bird remains squatting when approached or nudged. This criterion is assessed by approaching the bird, and if it remains squatting, gently nudging or touching the animal for 5 s. Animals may appear to rise but still rest upon their hocks. Only rising to stand on both feet within 5 s of handling is counted. A bird that takes longer than 5 s to rise, or that does not rise at all, is scored as 4. Nevertheless, the bird can walk when picked up by the observer and placed in a standing position, but squats immediately following one or 2 steps (Squatting often involves a characteristic ungainly backwards fall). Bird requires wings for balance.
5	Complete lameness	The bird cannot walk, and instead may shuffle along on its hocks. It may attempt to stand when approached but is unable to do so, and when placed on feet unable to complete a step with one or both legs.

**Table 3:** Descriptions of foot and toe abnormalities [31–33].

Type	Abnormality	Description
1	Shaky legs	Legs are shaking or quivering when the turkey is standing
2	Joint swelling	The hock joint is swollen
3	Valgus	Hocks are turned inward
4	Varus	Hocks are turned outward
5	Curl toes	Toes are curled inward or outward
6	Other (describe)	Any other abnormalities

### Postmortem footpad assessment

At 19 wk, a total of 144 birds (6 birds per pen) that represented the average body weight for birds in each pen were sent to the meat lab at Purdue university’s Land O’Lakes, Inc. Center for Experiential Learning for processing. At processing, footpad dermatitis was scored on a scale of 0 to 2 using the same scoring system as described in Table 1 to provide another method for scoring footpads when turkeys were immobilized and footpads were potentially easier to observe.

### Statistical analysis

All statistical analyses were performed using SAS software Version 9.4 (SAS Institute Inc., Cary, NC). Body weight differences among treatment groups and the interaction between treatment and age were analyzed using PROC GLIMMIX with pen as a random effect and age as a repeated measure, and Tukey’s test for multiple comparisons. Beak abnormalities and back wounds were not analyzed statistically because all turkeys received a score of 1 for beak (all turkeys were beak-trimmed but had no additional abnormalities) and all turkeys received a score of 0 for back wounds at all time points examined. Differences among treatments and ages in welfare scores were analyzed using PROC LOGISTIC with Firth bias-correction to correct for quasi-complete separation [34]. Pen was considered as the experimental unit. Feather quality on the wings was analyzed as binomial data because all turkeys received a score of either 1 or 2 across all time points. For scores that were collected from both wings and the feet, the highest score was used in the analysis. Data were initially examined to assess the frequencies for each score, and based on this assessment, the remaining welfare scores, postmortem footpad scores, and gait scores were analyzed as binomial data to compare good vs. affected (score 0 vs score > 0) due to the low numbers of turkeys in score categories 1 and higher; i.e. scores above 0 were combined into one category for analysis because of the small number of observations with score > 0. Room was considered as a covariate in the models for welfare scores and gait analysis. No interaction effects were found. Results were reported as odds ratio estimates with 95% confidence limits (CL) [35].

## Results

### Welfare assessment

#### Influence of environmental enrichment and age

No differences in body weight were found among treatment groups (Table 4). No back wounds or beak abnormalities (beyond trimming) were found across all observations in this study.

**Table 4:** Body weights (lsmean ± SE kg) of turkeys in each enrichment group at 8, 12, 16 and 19 weeks of age.

Group	8 wk	12 wk	16 wk	19 wk
**S**	3.94 ± 0.25	8.31 ± 0.28	14.24 ± 0.28	15.93 ± 0.25
**B**	4.19 ± 0.25	8.79 ± 0.28	14.95 ± 0.28	17.16 ± 0.25
**T**	4.15 ± 0.25	9.04 ± 0.28	14.44 ± 0.28	16.75 ± 0.25
**P**	3.82 ± 0.25	8.26 ± 0.28	14.49 ± 0.28	16.38 ± 0.25
**PS**	3.98 ± 0.25	8.66 ± 0.28	14.46 ± 0.28	16.86 ± 0.25
**C**	3.99 ± 0.25	8.73 ± 0.28	14.69 ± 0.28	16.78 ± 0.25

S = straw bale, B = pecking block, T = tunnel, P = platform, PS = platform + straw bale, C = control

No statistical differences were found among treatment groups at each age

All wing feather quality scores were found to be either a score of 1 or 2. Environmental enrichment significantly affected some turkey welfare measures, including feather quality on the neck (Wald *χ*^2^  = 11.305 P = 0.046), coverts (Wald *χ*^2^  = 30.27, P < 0.001) and wings (Wald *χ*^2^  = 19.437, P = 0.002). Environmental enrichment also affected feather cleanliness on the neck (Wald *χ*^2^ = 12.061, P = 0.034), back (Wald *χ*^2^   = 14.328, P = 0.014), rump (Wald *χ*^2^   = 26.815, P < 0.001), breast (Wald *χ*^2^   = 27.384, P < 0.001), belly (Wald *χ*^2^   = 23.54, P < 0.001), wings (Wald *χ*^2^   = 13.988, P = 0.016), and tail (Wald *χ*^2^   = 25.295, P < 0.001). Pairwise comparison results showed different patterns for the likelihood of having better welfare outcomes (fewer wounds, better feather quality, feather cleanliness, and footpad condition) among treatment groups (Table 5).

**Table 5:** Probability and odds ratio estimates for the overall influence of treatment on welfare measures.

Group	Wald *χ*^2^, Odds ratio estimate (95% CL) for having a lower score^1^										
	Neck FQ^2^	Coverts FQ^2^	Wings FQ^2^	Neck FC	Back FC	Rump FC	Breast FC	Belly FC	Wings FC^3^	Tail FC^3^	Footpad condition
**S vs. PS**	*χ*^2^ = 0.263 0.826 (0.398–1.715)	*χ*^2^ = 0.211 1.387 (0.344–5.601)	***χ***^**2**^ = 7.711 3.734 (1.473–9.463) **	*χ*^2^ = 0.435 0.351 (0.016–7.901)	*χ*^2^ = 1.684 3.336 (0.541–20.588)	*χ*^2^ = 0.551 3.287 (0.142–76.06)	***χ***^**2**^ = 11.734 2.943 (1.587–5.458) ***	***χ***^**2**^ = 7.700 2.755 (1.347–5.636) **	*χ*^2^ = 0.073 0.923 (0.515–1.654)	***χ***^**2**^ = 10.650 0.269 (0.122–0.592) **	*χ*^2^ = 0.726 1.474 (0.604–3.601)
**S vs. B**	*χ*^2^ = 0.099 1.116 (0.564–2.208)	***χ***^**2**^ = 13.478 8.273 (2.678–25.56) ***	***χ***^**2**^ = 10.600 5.804 (2.014–16.728) **	*χ*^2^ = 1.703 3.357 (0.545–20.687)	*χ*^2^ = 0.930 2.523 (0.385–16.555)	*χ*^2^ = 3.437 14.481 (0.858–244.4)	***χ***^**2**^ = 14.944 3.374 (1.821–6.25) ***	***χ***^**2**^ = 7.931 2.77 (1.363–5.63) **	*χ*^2^ = 1.496 1.435 (0.805–2.559)	*χ*^2^ = 1.474 0.592 (0.254–1.381)	*χ*^2^ = 3.796 2.297 (0.995–5.302)
**S vs. C**	*χ*^2^ = 2.944 0.489 (0.216–1.107)	***χ***^**2**^ = 4.903 3.836 (1.167–12.61) *	*χ*^2^ = 1.879 1.687 (0.799–3.563)	*χ*^2^ = 0.502 0.325 (0.014–7.302)	*χ*^2^ = 0.000 1.001 (0.109–9.193)	*χ*^2^ = 2.372 9.534 (0.541–168.1)	***χ***^**2**^ = 15.409 3.41 (1.848–6.291) ***	***χ***^**2**^ = 6.520 2.493 (1.237–5.025) *	***χ***^**2**^ = 5.893 2.056 (1.149–3.68) *	*χ*^2^ = 0.045 1.106 (0.435–2.809)	*χ*^2^ = 1.242 1.638 (0.688–3.901)
**S vs. P**	*χ*^2^ = 1.455 0.6208 (0.286–1.347)	***χ***^**2**^ = 5.815 4.277 (1.313–13.935) *	***χ***^**2**^ = 4.946 2.546 (1.117–5.803) *	***χ***^**2**^ = 4.818 6.815 (1.228–37.818) *	***χ***^**2**^ = 6.685 9.179 (1.71–49.272) **	***χ***^**2**^ = 7.515 47.575 (3.007–752.7) **	*χ*^2^ = 0.706 1.298 (0.706–2.387)	*χ*^2^ = 0.883 0.709 (0.346–1.452)	***χ***^**2**^ = 6.056 2.094 (1.162–3.773) *	*χ*^2^ = 2.075 2.325 (0.738–7.327)	*χ*^2^ = 1.041 1.581 (0.656–3.811)
**S vs. T**	***χ***^**2**^ = 6.543 0.279 (0.105–0.742) *	*χ*^2^ = 0.140 0.735 (0.146–3.696)	*χ*^2^ = 0.042 1.074 (0.54–2.139)	*χ*^2^ = 3.084 4.797 (0.834–27.607)	*χ*^2^ = 1.560 3.187 (0.517–19.651)	*χ*^2^ = 2.416 9.734 (0.552–171.6)	*χ*^2^ = 3.526 1.785 (0.975–3.269)	*χ*^2^ = 3.208 1.897 (0.941–3.821)	*χ*^2^ = 0.154 1.118 (0.629–1.99)	*χ*^2^ = 1.331 0.607 (0.26–1.417)	***χ***^**2**^ = 6.462 2.879 (1.274–6.505) *
**PS vs. B**	*χ*^2^ = 0.660 1.35 (0.654–2.787)	***χ***^**2**^ = 11.800 5.964 (2.153–16.523) ***	*χ*^2^ = 0.490 1.554 (0.452–5.344)	*χ*^2^ = 2.415 9.567 (0.554–165.1)	*χ*^2^ = 0.155 0.756 (0.188–3.041)	*χ*^2^ = 2.726 4.405 (0.758–25.608)	*χ*^2^ = 0.188 1.146 (0.618–2.126)	*χ*^2^ = 0.000 1.006 (0.493–2.051)	*χ*^2^ = 2.143 1.555 (0.861–2.809)	***χ***^**2**^ = 4.697 2.201 (1.078–4.49) *	*χ*^2^ = 1.241 1.558 (0.714–3.4)
**PS vs. C**	*χ*^2^ = 1.454 0.592 (0.253–1.388)	*χ*^2^ = 3.364 2.766 (0.933–8.203)	*χ*^2^ = 2.508 0.452 (0.169–1.208)	*χ*^2^ = 0.002 0.926 (0.021–41.674)	*χ*^2^ = 1.680 0.3 (0.049–1.853)	*χ*^2^ = 1.301 2.9 (0.466–18.07)	*χ*^2^ = 0.221 1.159 (0.627–2.141)	*χ*^2^ = 0.077 0.905 (0.447–1.833)	***χ***^**2**^ = 6.974 2.229 (1.23–4.04) **	***χ***^**2**^ = 11.603 4.113 (1.823–9.28) ***	*χ*^2^ = 0.064 1.111 (0.492–2.507)
**PS vs. P**	*χ*^2^ = 0.476 0.752 (0.334–1.692)	***χ***^**2**^ = 4.197 3.084 (1.05–9.057) *	*χ*^2^ = 0.519 0.682 (0.241–1.932)	***χ***^**2**^ = 4.366 19.4249 (1.2020–313.9) *	*χ*^2^ = 3.223 2.751 (0.911–8.305)	***χ***^**2**^ = 10.020 14.472 (2.767–75.706) **	***χ***^**2**^ = 6.698 0.441 (0.237–0.82) **	***χ***^**2**^ = 12.984 0.257 (0.123–0.539) ***	***χ***^**2**^ = 7.139 2.27 (1.244–4.141) **	***χ***^**2**^ = 16.099 8.648 (3.015–24.805) ***	*χ*^2^ = 0.028 1.072 (0.469–2.451)
**PS vs. T**	***χ***^**2**^ = 4.459 0.338 (0.123–0.925) *	*χ*^2^ = 0.653 0.53 (0.113–2.474)	*χ*^2^ = 6.745 0.288 (0.112–0.737) **	*χ*^2^ = 3.340 13.674 (0.827–226)	*χ*^2^ = 0.005 0.955 (0.259–3.528)	*χ*^2^ = 1.352 2.961 (0.475–18.454)	*χ*^2^ = 2.552 0.607 (0.329–1.12)	*χ*^2^ = 1.066 0.688 (0.339–1.399)	*χ*^2^ = 0.410 1.212 (0.673–2.183)	***χ***^**2**^ = 5.020 2.259 (1.107–4.61) *	*χ*^2^ = 2.999 1.953 (0.916–4.164)
**B vs. C**	***χ***^**2**^ = 3.964 0.439 (0.195–0.987) *	***χ***^**2**^ = 4.490 0.464 (0.228–0.944) *	***χ***^**2**^ = 4.796 0.291 (0.096–0.878) *	*χ*^2^ = 2.586 0.097 (0.006–1.667)	*χ*^2^ = 0.927 0.397 (0.06–2.606)	*χ*^2^ = 0.459 0.658 (0.196–2.208)	*χ*^2^ = 0.001 1.011 (0.549–1.861)	*χ*^2^ = 0.088 0.9 (0.447–1.81)	*χ*^2^ = 1.437 1.433 (0.796–2.581)	*χ*^2^ = 1.984 1.869 (0.783–4.464)	*χ*^2^ = 0.778 0.713 (0.337–1.512)
**B vs. P**	*χ*^2^ = 2.231 0.557 (0.258–1.201)	*χ*^2^ = 3.353 0.517 (0.258–1.037)	*χ*^2^ = 1.946 0.439 (0.138–1.396)	*χ*^2^ = 1.431 2.03 (0.636–6.48)	***χ***^**2**^ = 4.425 3.638 (1.092–12.115) *	***χ***^**2**^ = 6.364 3.285 (1.304–8.278) *	***χ***^**2**^ = 9.166 0.385 (0.207–0.714) **	***χ***^**2**^ = 13.321 0.256 (0.123–0.532) ***	*χ*^2^ = 1.552 1.46 (0.805–2.646)	***χ***^**2**^ = 5.966 3.93 (1.311–11.785) *	*χ*^2^ = 0.916 0.688 (0.32–1.479)
**B vs. T**	***χ***^**2**^ = 7.781 0.25 (0.095–0.662) **	***χ***^**2**^ = 13.284 0.089 (0.024–0.327) ***	***χ***^**2**^ = 9.588 0.185 (0.064–0.538) **	*χ*^2^ = 0.332 1.429 (0.424–4.813)	*χ*^2^ = 0.108 1.263 (0.314–5.074)	*χ*^2^ = 0.414 0.672 (0.2–2.255)	***χ***^**2**^ = 4.166 0.529 (0.287–0.975) *	*χ*^2^ = 1.119 0.685 (0.339–1.381)	*χ*^2^ = 0.702 0.779 (0.435–1.397)	*χ*^2^ = 0.004 1.027 (0.472–2.235)	*χ*^2^ = 0.412 1.253 (0.629–2.496)
**C vs. P**	*χ*^2^ = 0.276 1.269 (0.521–3.09)	*χ*^2^ = 0.072 1.115 (0.505–2.463)	*χ*^2^ = 0.834 1.51 (0.624–3.653)	***χ***^**2**^ = 4.602 20.989 (1.301–338.8) *	***χ***^**2**^ = 6.668 9.17 (1.706–49.296) **	***χ***^**2**^ = 8.996 4.99 (1.746–14.266) **	***χ***^**2**^ = 9.489 0.381 (0.206–0.704) **	***χ***^**2**^ = 11.590 0.285 (0.138–0.587) ***	*χ*^2^ = 0.004 1.019 (0.56–1.851)	*χ*^2^ = 1.564 2.103 (0.656–6.739)	*χ*^2^ = 0.008 0.965 (0.434–2.147)
**C vs. T**	*χ*^2^ = 1.053 0.571 (0.196–1.666)	***χ***^**2**^ = 5.693 0.192 (0.049–0.744) *	*χ*^2^ = 1.352 0.637 (0.298–1.362)	*χ*^2^ = 3.544 14.775 (0.895–243.8)	*χ*^2^ = 1.555 3.184 (0.516–19.653)	*χ*^2^ = 0.001 1.021 (0.276–3.78)	***χ***^**2**^ = 4.361 0.524 (0.285–0.961) *	*χ*^2^ = 0.595 0.761 (0.38–1.524)	***χ***^**2**^ = 4.143 0.544 (0.303–0.978) *	*χ*^2^ = 1.822 0.549 (0.23–1.311)	*χ*^2^ = 2.310 1.757 (0.849–3.636)
**P vs. T**	*χ*^2^ = 2.272 0.45 (0.159–1.271)	***χ***^**2**^ = 6.642 0.172 (0.045–0.663) *	*χ*^2^ = 4.100 0.422 (0.183–0.973) *	*χ*^2^ = 0.430 0.704 (0.246–2.011)	*χ*^2^ = 3.527 0.347 (0.115–1.047)	***χ***^**2**^ = 8.758 0.205 (0.072–0.585) **	*χ*^2^ = 1.054 1.375 (0.749–2.527)	***χ***^**2**^ = 7.137 2.675 (1.3–5.504) **	***χ***^**2**^ = 4.297 0.534 (0.295–0.966) *	***χ***^**2**^ = 5.743 0.261 (0.087–0.783) *	*χ*^2^ = 2.508 1.821 (0.867–3.822)

S = straw bale, B = pecking block, T = tunnel, P = platform, PS = platform + straw bale, C = control, FQ = feather quality, FC = feather cleanliness

Back wounds were assessed; however, all birds had a score of 0. No difference was found for head, neck or tail wounds.

^1^ * P < 0.05,

** P < 0.01,

*** P < 0.001;

^2^ No difference was found on feather quality on back, rump or tail.

^3^ Feather cleanliness on wings and tail were only assessed for 16 vs. 19 wk.

There were some age-related differences in welfare measures that were unique to some treatment groups. Specifically, P turkeys were 19.159 times more likely to have better neck feather cleanliness at 8 wk than at 19 wk (Wald *χ*^2^ = 4.084; 95% CL 1.093–335.8; P = 0.043), and 7.128 times more likely to have better back feather cleanliness at 8 wk than at 19 wk (Wald *χ*^2^ = 4.699; 95% CL 1.207–42.088; P = 0.03). Rump feather cleanliness was better at 8, 12 and 19 wk compared to 16 wk for P turkeys (8 vs. 16 wk: Wald *χ*^2^ = 9.052; 79.556, 4.597–1376.7; P = 0.003; 12 vs. 16 wk: Wald *χ*^2^ = 6.276; 39.815, 2.23–710.9; P = 0.012; 16 vs. 19 wk: Wald *χ*^2^ = 8.997; 0.191, 0.065–0.564; P = 0.003. In the B group, tail feather cleanliness was 4.709 times more likely to be better at 16 wk than at 19 wk (Wald *χ*^2^ = 15.311; 95% CL 2.167–10.232; P < 0.001). In the T group, turkeys were 20.912 times more likely to have fewer head wounds at 8 wk than at 16 wk (Wald *χ*^2^ = 4.120; 95% CL 1.11–394; P = 0.042).

Age significantly affected the majority of turkey welfare measures:

1. wounds (head: Wald *χ*^2^  = 8.529, P = 0.036; neck: Wald *χ*^2^  = 13.486, P = 0.004; tail: Wald *χ*^2^  = 9.791, P = 0.02),
2. feather quality (neck: Wald *χ*^2^   = 34.725, P < 0.001; back: Wald *χ*^2^   = 8.409, P = 0.038; rump: Wald *χ*^2^   = 11.881, P = 0.008; coverts: Wald *χ*^2^  = 10.811, P = 0.013; wings: Wald *χ*^2^  = 28.788, P < 0.001),
3. feather cleanliness (neck: Wald *χ*^2^   = 15.411, P = 0.002; back: Wald *χ*^2^   = 20.19, P < 0.001; rump: Wald *χ*^2^  = 13.967, P = 0.003; breast: Wald *χ*^2^   = 186.261, P < 0.001; belly: Wald *χ*^2^  = 214.396, P < 0.001; wings: Wald *χ*^2^  = 43.474, P < 0.001; tail: Wald *χ*^2^  = 6.184, P = 0.013), and
4. footpad condition (Wald *χ*^2^   = 62.379, P < 0.001).

#### Age-related changes in welfare measures within each treatment group

Within treatment groups, various welfare measures changed with age, and changes in feather quality of the neck and wings, footpad condition and feather cleanliness of the breast, belly and wings were similar for several treatment groups.

Neck feather quality was worse at 19 wk than at earlier time points for turkeys in S and C groups. Turkeys in the S group were more likely to have better feather quality at 8 (odds ratio and 95% CL: 15.730; 2.804–88.271; Wald *χ*^2^ = 9.808; P = 0.002), 12 (25.600; 1.441–454.8; Wald *χ*^2^ = 4.879; P = 0.027) and 16 (8.210; 1.413–47.704; Wald *χ*^2^ = 5.50; P = 0.019) wk than at 19 wk. Neck feather quality was worse at 16 wk than at other time points for turkeys in P and PS groups. Turkeys in the P group were 5.085 times more likely to have better neck feather quality at 8 wk than at 16 wk (95% CL 1.078–23.982; Wald *χ*^2^ = 4.222; P = 0.0400). Similarly, Turkeys in the PS group were 38.638 times more likely to have better neck feather quality at 8 wk (95% CL 2.168–688.5; Wald *χ*^2^ = 6.183; P = 0.013), and 19.706 times more likely at 12 wk (95% CL 1.069–363.3; Wald *χ*^2^ = 4.019; P = 0.045) than at 16 wk.

Wing feather quality improved with age for turkeys in S and T groups. In the S group, turkeys at 8 wk were less likely to have better wing feather quality than at 16 wk (0.036; 95% CL 0.002–0.702; Wald *χ*^2^ = 4.807; P = 0.028), and 19 wk (0.023; 95% CL 0.001–0.416; Wald *χ*^2^ = 6.526; P = 0.011). Similarly, turkeys in the T group were less likely to have better wing feather quality at 8 wk than at 19 wk (0.145; 95% CL 0.035–0.61; Wald *χ*^2^ = 6.944; P = 0.008).

Footpad condition worsened over time for turkeys in all treatment groups except for the S group. Turkeys in the P (26.116; 1.543–441.9; Wald *χ*^2^ = 5.111; P = 0.024), PS (19.947; 1.161–342.6; Wald *χ*^2^ = 4.256; P = 0.039), B (39.573; 2.334–670.9; Wald *χ*^2^ = 6.487; P = 0.011), T (66.165; 3.947–1109.3; Wald *χ*^2^ = 8.493; P = 0.004) and C (34.372; 2.051–576.2; Wald *χ*^2^ = 6.048; P = 0.014) groups were more likely to have better footpad condition at 8 wk than at 19 wk. Turkeys in the B (20.721; 1.185–362.3; Wald *χ*^2^ = 4.311; P = 0.038), T (34.674; 2.004–599.8; Wald *χ*^2^ = 5.944; P = 0.015), and C (18.351; 1.062–317.2; Wald *χ*^2^ = 4.005; P = 0.045) groups were more likely to have better footpad condition at 12 wk than at 19 wk. In addition, turkeys in the T (10.994; 1.947–62.077; Wald *χ*^2^ = 7.369; P = 0.007) and C (5.829; 1.018–33.371; Wald *χ*^2^ = 3.921; P = 0.048) groups were more likely to have better footpad condition at 16 wk than at 19 wk. Footpad condition was better at 8 than at 16 wk for turkeys in the B group (19.97; 1.052–379; Wald *χ*^2^ = 3.976; P = 0.046).

Results from postmortem assessment of footpad dermatitis were consistent with results collected prior to processing. Turkeys in the S group had the lowest prevalence of footpad dermatitis compared to P (15.463; 3.656–65.395; Wald *χ*^2^ = 13.855; P < 0.001), PS (7.134; 1.814–28.063; Wald *χ*^2^ = 7.908; P = 0.005), B (8.537; 2.148–33.938; Wald *χ*^2^ = 9.275; P = 0.002), T (19.501; 4.443–85.585; Wald *χ*^2^ = 15.494; P < 0.001), and C (34.607; 6.904–173.5; Wald *χ*^2^ = 18.568; P < 0.001) turkeys. PS turkeys were also more likely to have better footpad condition compared to C turkeys (4.851; 1.139–20.653; Wald *χ*^2^ = 4.564; P = 0.033).

Breast feather cleanliness was better at 8 and 12 wk than at 19 wk for turkeys in all treatment groups (Table 6). Turkeys in S, P T and C groups also had cleaner feathers at 16 vs. 19 wk. Breast feather cleanliness was better at 8 versus 16 wk in PS, B, T and C groups. PS, B and C turkeys also had cleaner feathers at 12 versus 16 wk. In addition, P turkeys had better breast cleanliness at 8 versus 12 wk. Similar to breast feather cleanliness, belly feather cleanliness was better at 8, 12 and 16 wk than at 19 wk in all treatment groups. Belly feather cleanliness was also better at 8 and 12 versus 16 wk in PS, B and C groups. Wing feather cleanliness was better at 16 versus 19 wk in S, B, T and C groups. Pairwise comparison results of breast, belly and wing feather cleanliness are presented in Table 6.

**Table 6:** Probability and odds ratio estimates for significant age-related changes in feather cleanliness within each treatment group.

Group	Age (wk)	Wald *χ*^2^, Odds ratio estimate (95% CL) of having better condition		
		Breast feather cleanliness	Belly feather cleanliness	Wing feather cleanliness
**S**	**8 vs. 19**	*χ*^2^ = 3.111	*χ*^2^ = 17.792	
		205.7 (12.08–3501.2) ***	4823.2 (93.718–248224) ***	
	**12 vs. 19**	*χ*^2^ = 17.149	*χ*^2^ = 16.478	
		13.861 (3.994–48.104) ***	815.2 (32.034–20747.3) ***	
	**16 vs. 19**	*χ*^2^ = 10.225	*χ*^2^ = 14.981	*χ*^2^ = 4.113
		107.5 (6.113–1889.2) **	2585.8 (48.365–138243) ***	1.557 (1.015–2.39) *
**P**	**8 vs. 12**	*χ*^2^ = 4.007		
		20.91 (1.066–410.4) *		
	**8 vs. 19**	*χ*^2^ = 15.179	*χ*^2^ = 18.013	
		303.7 (17.124–5385.9) ***	505.6 (126.245–9741.6) ***	
	**12 vs. 19**	*χ*^2^ = 18.309	*χ*^2^ = 20.987	
		14.524 (4.264–49.473) ***	44.811 (8.808–228) ***	
	**16 vs. 19**	*χ*^2^ = 17.514	*χ*^2^ = 12.906	
		47.476 (7.786–289.5) ***	248.8 (12.266–5045.1) ***	
**PS**	**8 vs. 16**	*χ*^2^ = 11.836	*χ*^2^ = 10.599	
		154.9 (8.758–2740.9) ***	122.8 (6.785–2224.4) **	
	**12 vs. 16**	*χ*^2^ = 12.360	*χ*^2^ = 9.999	
		25.449 (4.188–154.6) ***	11.709 (2.548–53.804) **	
	**8 vs. 19**	*χ*^2^ = 15.077	*χ*^2^ = 16.647	
		276.5 (16.187–4721.2) ***	394.6 (22.338–6970.7) ***	
	**12 vs. 19**	*χ*^2^ = 18.318	*χ*^2^ = 23.210	
		45.407 (7.911–260.6) ***	37.611 (8.599–164.5) ***	
	**16 vs. 19**		*χ*^2^ = 5.948	
			3.212 (1.258–8.205) *	
**B**	**8 vs. 16**	*χ*^2^ = 13.959	*χ*^2^ = 10.761	
		242.6 (13.607–4324.2) ***	139.6 (7.301–2669.1) **	
	**12 vs. 16**	*χ*^2^ = 10.671	*χ*^2^ = 7.852	
		128.8 (6.983–2376.6) **	72.762 (3.627–1459.6) **	
	**8 vs. 19**	*χ*^2^ = 15.582	*χ*^2^ = 22.654	
		307 (17.874–5272.3) ***	1896.6 (84.748–42443.6) ***	
	**12 vs. 19**	*χ*^2^ = 12.033	*χ*^2^ = 18.380	
		163 (9.168–2899) ***	988.6 (42.244–23133.3) ***	
	**16 vs. 19**		*χ*^2^ = 13.368 13.586 (3.355–55.017) ***	*χ*^2^ = 10.442 2.101 (1.339–3.296) **
**T**	**8 vs. 16**	*χ*^2^ = 4.048		
		21.668 (1.083–433.7) *		
	**8 vs. 19**	*χ*^2^ = 19.970	*χ*^2^ = 21.809	
		1029.8 (49.123–21588.3) ***	1570 (71.55–34448.3) ***	
	**12 vs. 19**	*χ*^2^ = 15.935	*χ*^2^ = 17.780	
		537.9 (24.544–11787.7) ***	837.9 (36.679–19141.2) ***	
	**16 vs. 19**	*χ*^2^ = 23.583	*χ*^2^ = 17.543	*χ*^2^ = 18.590
		47.527 (10.003–225.8) ***	796.3 (34.957–18140.1) ***	3.555 (1.997–6.328) ***
**C**	**8 vs. 16**	*χ*^2^ = 10.816	*χ*^2^ = 10.542	
		122.2 (6.971–2143.3) **	129.3 (6.869–2433.6) **	
	**12 vs. 16**	*χ*^2^ = 8.00	*χ*^2^ = 7.772	
		65.275 (3.606–1181.6) **	69.224 (3.519–1361.6) **	
	**8 vs. 19**	*χ*^2^ = 18.283	*χ*^2^ = 20.501	
		502 (29.024–8682.7) ***	986.9 (49.903–19517.8) ***	
	**12 vs. 19**	*χ*^2^ = 14.454	*χ*^2^ = 16.475	
		268.1 (15.012–4787.1) ***	528.4 (25.592–10909.2) ***	
	**16 vs. 19**	*χ*^2^ = 9.426	*χ*^2^ = 12.353	*χ*^2^ = 11.896
		4.107 (1.667–10.12) **	7.633 (2.457–23.71) **	2.184 (1.401–3.404) ***

S = straw bale, B = pecking block, T = tunnel, P = platform, PS = platform + straw bale, C = control

* P < 0.05,

** P < 0.01,

*** P < 0.001

### Gait assessment and foot and toe abnormalities

The percentage of turkeys/treatment group with each gait score is presented in Table 7. Age significantly affected gait scores of the birds (Wald *χ*^2^  = 209.806, P < 0.001) while treatment had no overall effect on gait scores. Gait worsened with increasing age in all treatment groups. Gait was worst in the S (Wald *χ*^2^  = 36.455, P < 0.001), P (Wald *χ*^2^  = 40.357, P < 0.001), PS (Wald *χ*^2^  = 47.698, P < 0.001) and B (Wald *χ*^2^  = 39.091, P < 0.001) groups at 19 wk compared to 8, 12 and 16 wk, while gait in T (Wald *χ*^2^  = 38.166, P < 0.001) and C (Wald *χ*^2^  = 38.129, P < 0.001) groups worsened starting at 16 wk (Table 8).

**Table 7:** Percentage of turkeys in each treatment group with each gait score at 8, 12, 16, and 19 wk.

Group	Age (wk)	Gait score					
		0	1	2	3	4	5
**S**	**8**	100	0	0	0	0	0
	**12**	100	0	0	0	0	0
	**16**	95.59	4.41	0	0	0	0
	**19**	56.72	41.79	1.49	0	0	0
**PS**	**8**	98.46	0	0	1.54	0	0
	**12**	100	0	0	0	0	0
	**16**	90.48	9.52	0	0	0	0
	**19**	44.44	47.62	7.94	0	0	0
**B**	**8**	100	0	0	0	0	0
	**12**	100	0	0	0	0	0
	**16**	93.85	6.15	0	0	0	0
	**19**	51.56	37.5	10.94	0	0	0
**C**	**8**	98.53	0	0	0	1.47	0
	**12**	100	0	0	0	0	0
	**16**	88.06	11.94	0	0	0	0
	**19**	53.03	42.42	4.55	0	0	0
**P**	**8**	100	0	0	0	0	0
	**12**	100	0	0	0	0	0
	**16**	92.31	7.69	0	0	0	0
	**19**	49.23	44.62	6.15	0	0	0
**T**	**8**	100	0	0	0	0	0
	**12**	98.48	0	1.52	0	0	0
	**16**	87.88	10.61	0	0	1.52	0
	**19**	52.31	41.54	6.15	0	0	0

S = straw bale group, B = pecking block group, T = tunnel group, P = platform group, PS = platform + straw bale group, C = control group

**Table 8:** Probability and odds ratio estimates for age-related changes in gait score within each treatment group.

Group	Wald *χ*^2^, Odds ratio estimate (95% CL) of having better condition^1^					
	8 vs. 12 wk	8 vs. 16 wk	8 vs. 19 wk	12 vs. 16 wk	12 vs. 19 wk	16 vs. 19 wk
**S**	*χ*^2^ = 0.0 1.009 (0.02–51.157)	*χ*^2^ = 1.741 7.421 (0.378–145.8)	***χ***^**2**^ = 10.666 110.1 (6.554–1850.9) **	*χ*^2^ = 1.724 7.356 (0.374–144.6)	***χ***^**2**^ = 10.618 109.2 (6.489–1836.3) **	***χ***^**2**^ = 19.719 14.8407 (4.512–48.809) ***
**PS**	*χ*^2^ = 0.449 0.334 (0.013–8.28)	*χ*^2^ = 2.925 4.845 (0.794–29.547)	***χ***^**2**^ = 21.072 52.391 (9.665–284.0) ***	*χ*^2^ = 3.288 14.517 (0.805–261.7)	***χ***^**2**^ = 12.354 157 (9.362–2632.5) ***	***χ***^**2**^ = 24.025 10.8145 (4.174–28.02) ***
**B**	*χ*^2^ = 0.0 1.019 (0.02–51.636)	*χ*^2^ = 2.342 9.901 (0.525–186.6)	***χ***^**2**^ = 11.428 129.9 (7.730–2183.3) ***	*χ*^2^ = 2.302 9.713 (0.515–183.1)	***χ***^**2**^ = 11.338 127.4 (7.583–2142) ***	***χ***^**2**^ = 21.627 13.1214 (4.434–38.829) ***
**P**	*χ*^2^ = 0.0 1.03 (0.02–51.979)	*χ*^2^ = 2.890 12.434 (0.68–227.4)	***χ***^**2**^ = 11.80 138.7 (8.312–2315.8) ***	*χ*^2^ = 2.819 12.071 (0.659–221)	***χ***^**2**^ = 11.647 134.7 (8.062–2250.2) ***	***χ***^**2**^ = 22.353 11.1582 (4.105–30.331) ***
**T**	*χ*^2^ = 0.472 3.084 (0.124–76.699)	***χ***^**2**^ = 4.121 19.5311 (1.108–344.3) *	***χ***^**2**^ = 11.137 121.7 (7.254–2041.6) ***	***χ***^**2**^ = 4.173 6.333 (1.078–37.214) *	***χ***^**2**^ = 18.176 39.4592 (7.284–213.8) ***	***χ***^**2**^ = 16.914 6.2307 (2.606–14.9) ***
**C**	*χ*^2^ = 0.461 0.329 (0.013–8.161)	***χ***^**2**^ = 4.231 6.340 (1.091–37.530) *	***χ***^**2**^ = 18.187 39.2273 (7.264–211.8) ***	***χ***^**2**^ = 4.117 19.4726 (1.106–342.8) *	***χ***^**2**^ = 11.063 119.4 (7.129–1998.4) ***	***χ***^**2**^ = 16.783 6.1294 (2.575–14.593) ***

S = straw bale group, PS = platform + straw bale group, B = pecking block group, P = platform group, T = tunnel group, C = control group

^1^ * P < 0.05,

** P < 0.01,

*** P < 0.001

Few abnormalities of the feet and toes were observed across all ages. In the S group, three birds had joint swelling (1 at 16 wk and 2 at 19 wk). In the PS group, one bird had shaky legs at 16 wk, three birds had joint swelling, one bird had a valgus deformity, one bird had a varus deformity, and three birds had curl toes at 19 wk. In the B group, three birds had curl toes (1 at 16 wk and 2 at 19 wk), one bird had joint swelling, and three birds had a valgus deformity at 19 wk. In the C group, one bird had shaky legs at 16 wk, two birds each had a swollen toe at 19 wk, and three birds had curl toes (2 at 16 wk and 1 at 19 wk). In the P group, six birds had joint swelling (3 at 16 wk and 3 at 19 wk), two birds had shaky legs, and one bird had a valgus deformity at 19 wk. In the T group, two birds had shaky legs at 16 wk, four birds had joint swelling, and three birds had curl toes at 19 wk.

## Discussion

To the best of our knowledge, this is the first study to examine the effects of different types of environmental enrichment on gait and age-related changes in turkey welfare measures and walking ability. Our results indicate that age had an overall effect on the majority of welfare measures, and different enrichments had an overall effect mostly on feather quality and feather cleanliness. Compared to earlier ages, neck feather quality was worst at 19 wk in the straw bale and control groups, while worst at 16 wk then improved at 19 wk in the platform and platform + straw bale groups. Wing feather quality improved with age in the straw bale and tunnel groups. Footpad condition worsened over time for turkeys in all treatment groups except for the straw bale group. Breast, belly and wing feather cleanliness worsened with age in all treatment groups. Gait was worst at 19 wk compared to all other time points in the straw bale, platform, straw bale + platform and pecking block groups, whereas gait started worsening for birds in the tunnel and control groups at 16 wk. Feet and toe abnormalities were observed infrequently.

### Influence of EE on welfare measures

#### Feather condition

Neck feather damage can often be caused by aggressive pecking, which is specifically aimed at the head and neck areas [36] and can also be caused by feather pecking [37]. A previous study reported that injurious pecking on the head and neck areas often originated from fights in small turkey flocks as turkeys attempt to establish their social hierarchy, which begins around 8 wk [38]. Previous research demonstrated that the provision of an elevated platform structure (‘turkey tree’) as enrichment was beneficial in reducing feather pecking and fighting and resulted in improved feather quality [22]. Our results agree with those of Lindenwald et al. [22]; turkeys provided with platforms or platforms and straw bales had improved feather condition compared to turkeys in other treatment groups. The platforms in this study provided turkeys with additional space and areas to potentially avoid or escape injurious pecking and aggression. Although straw bales can also serve as elevated structures, the bales provided limited space for turkeys to avoid aggressive conspecifics compared to the elevated platform, because turkeys provided with platforms had access to the top, as well as to areas underneath the platform and ramps. However, use of the space underneath the platform and ramps was not systematically assessed, so the extent to which turkeys used this space is unknown.

Our observations of better wing feather quality with age for turkeys in the straw bale group were consistent with findings of Martrenchar et al. [20] who reported reduced wing damage in birds provided with straw bales. Straw bales are one of the primary enrichments recommended for turkeys, as they can satisfy turkeys’ pecking and foraging needs [14]. Turkeys provided with a tunnel as enrichment had better wing feather quality at 19 wk. The tunnel was designed to give turkeys a secluded rest area as well as an opportunity to hide from and avoid their conspecifics, as tunnel walls can serve similar functions to visual barriers (e.g. free-standing wooden boards). Results from the tunnel group were consistent with those of Sherwin et al. [18], who reported that visual barriers can help reduce wing and tail pecking injuries in turkeys. Visual barriers are listed as a secondary enrichment to provide turkeys with a hiding structure in the G.A.P. standards [14]. Anecdotally, we also observed turkeys resting on top of the tunnel at young ages, indicating that the tunnel may also serve as an elevated space, at least when turkeys are young. It is important to note, however, that tunnels had to be reinforced with a second corrugated plastic sheet as turkeys grew heavier because the tunnels became flattened as the turkeys continued to attempt to rest on them.

When examining feather condition, it is important to consider when turkey feathers molt, because feather development and molting may also influence turkey feather condition, and hence, how feather condition is scored in welfare assessments. Juvenile turkeys usually start to molt their primary wing feathers at 5 wk, and molting continues approximately every week until 10 wk when the molting interval slows to every 2 to 3 weeks until 20 wk (later ages not observed) [39]. Turkeys begin to molt their body plumage at 7 wk, starting across the breast, then continuing to the back and belly, and lastly the neck. At 14 wk, new feathers often appear on the neck and the post juvenal feathers are mostly fully developed on all body regions except the head [40]. In our study, neck feather condition was observed to be worst after 16 wk, which is after neck feather molting would have been completed. Wing feather condition was observed to worsen from 8 wk until the end of the study at 19 wk, which coincided with the molting of wing feathers. However, feather condition scored as 1 or 2 in this study was due to broken feathers (the ends of feathers were damaged or broken), likely caused by feather pecking, and not molting (bald patches with newly emerging feathers).

#### Footpad condition

The worst footpad condition was observed at 19 wk in the platform, platform + straw bale, tunnel and control groups while footpad condition for turkeys in the pecking block group worsened from 16 wk. The pecking blocks were donated and were similar to other commercially available pecking blocks, but detailed information about the contents of the pecking blocks were proprietary. It is possible that the contents of the pecking blocks could have changed litter moisture content, if for example, the pecking blocks resulted in birds drinking more water or resulted in changes in birds’ excreta relative to the other treatment groups. A limitation of this study is that we were not able to determine whether pecking blocks influenced litter quality, and additional research will be valuable in providing information about the influence of pecking blocks on litter condition and footpad condition. Footpad condition did not worsen with age for turkeys provided with a straw bale. Postmortem assessment of footpad dermatitis confirmed that the straw bale group had the best footpad condition and the platform + straw bale had better footpad condition than the control group. Footpad condition is affected by multiple factors including litter condition, environmental conditions (e.g. temperature and humidity), drinker design, stocking density, body weight, group size and diet etc. [2]. The most influential factor causing footpad dermatitis is high litter moisture; the exposure of turkeys to wet litter without feces and urine for 8 hr/day was sufficient to cause bad footpad lesions [41]. The bell drinker used in this study was observed to be bumped by turkeys and water spills happened on several occasions. Attempts were made to add fresh, dry shavings when spills occurred, but wet litter because of water spills likely resulted in the worsening footpad condition observed in this study. The worse footpad condition observed at later ages may also be related to turkeys’ larger body size and higher body weight, which result in both increased area of contact surface and higher contact pressure of footpads with the litter [13]. Straw bales may have been beneficial in reducing turkeys’ contact with wet litter because straw is not as absorptive as wood shavings. Loose straw pulled out from the straw bales may have served as a barrier between turkeys’ feet and the wet litter so that worse footpad condition was not observed in the straw bale group. Footpad condition of turkeys in the platform + straw bale group was slightly worse than turkeys provided only with a bale, as the platform occupied a large amount of the space in the pen and may have limited the floor space over which loose straw could be scattered. However, the platform + straw bale group still had a lower prevalence of footpad dermatitis compared to the control group. Previous studies have not examined the influence of environmental enrichment on turkeys’ footpad condition and further work is needed to examine the interactive effects among litter management, litter condition, environmental enrichment and footpad condition.

### Influence of EE on gait score

Previous studies have linked rapid weight gain and exacerbated growth of the breast muscle with gait impairments and reduced walking ability in turkeys [2, 12, 13]. However, limited research to date has examined the influence of environmental enrichment on turkey walking ability. One study, Sherwin et al. [21], reported potentially improved skeletal health in turkeys provided with various types of enrichment (wooden board with rope and screws attached, reflective sheeting sandwiched between clear acrylic sheets, cabbages, and supplemental spotlights that turned on and off). Other studies with broiler chickens have suggested a link between environmental enrichment, activity level, and walking ability. Better walking ability (better gait) for broilers with access to platforms was reported by Kaukonen et al. [42], and they suggested that birds benefited from the locomotor activity required to use the platform. Similarly, Bailie et al. [43] reported that lameness was worse for broiler chickens that had not been provided with a straw bale compared to broilers that had access to a straw bale, and Bizeray et al. [44] suggested that providing wood barriers increased broilers’ activity levels. Enrichment can influence both activity level and body weight, which could impact walking ability. Body weight did not differ among treatment groups in our study, and while we found no overall effect of enrichment type on gait score of turkeys, the finding that gait worsened at 16 wk for turkeys in the control and tunnel groups, compared to gait worsening at 19 wk for turkeys provided with a straw bale, platform, straw bale and platform or pecking block suggests that there may be some beneficial effects of enrichment on gait that warrants further investigation. Both pecking and foraging enrichment (straw bale or pecking block) and elevated structures (straw bale or platform) can provide opportunities for turkeys to perform more activities and more locomotion compared to control conditions. Stocking density is another factor that could influence gait scores. The enrichments used in this study all occupied relatively different amounts of space, and altered the spatial configuration of the pens as well as the total amount of space available to the turkeys (and hence the stocking density). It is possible that the amount of available space may alter gait independently of the enrichment used, but all of our enrichments were very different and it was not possible to control stocking density. For example, turkeys provided with straw bales could perch on top of the bale, and had a similar overall amount of space as turkeys in the control group. Turkeys provided with a platform had the additional platform space available, but the ramps took up space and therefore reduced available floor space. Additionally, the relatively small numbers of turkeys in each pen meant that if there was mortality in some pens, this would also alter the stocking density, and we did not cull birds to maintain the exact same group sizes in each pen. Since this study aimed to examine how turkeys used different enrichments within the constraints of relatively small pen sizes, controlling the amount of floor space was not feasible. Enrichments also change the quality of the environment, not just the space available; therefore, there are other factors (how turkeys use the space) that need to be considered in addition to floor space. In commercial turkey production, producers that follow animal welfare certification programs have a choice of several different types of enrichment (e.g. Global Animal Partnership’s 5-step Program [14]). These enrichments, when applied in a commercial context, would similarly change the space available in the barn, and some enrichments may increase stocking density whereas others may provide additional useable space. Further work is needed to clearly establish whether these enrichments or the amount or type of space available in a pen influence gait and activity level.

### Age-related changes in welfare measures and gait

#### Feather condition

In previous studies, age was reported to affect turkey feather condition. For example, Duggan et al. [5] reported that the average feather condition of the neck, back, wings, and tail improved from 9 to 15 wk (no observations at later ages) in turkeys housed in entirely enclosed commercial barns, whereas feather condition was worst at 15 wk in turkeys housed in curtain-sided commercial barns. Others have reported that feather pecking became more prevalent with age [45] and the incidence of feather pulls increased with age from 3 to 9 wk [4]. In the present study, wing feather quality improved with age in straw bale and tunnel groups, and neck feather quality was better from 16 to 19 wk in platform and platform + straw bale groups. Our findings differ from results reported by Duggan et al., [5] who collected data from commercial farms. However, group size, stocking density and environmental conditions were different between the two studies and a direct comparison of results is not possible (group size: 16–18 birds per pen in this study vs. around 4800 birds per barn in Duggan et al. [5]; stocking density: 2.15 to 2.42 birds/m^2^ in this study vs. 2.56 to 2.78 birds/m^2^ in Duggan et al. [5]). Further work is needed to determine the influence of environmental enrichment on feather condition. In particular, it will be important to examine whether feather pecking behavior differs among turkeys provided with different types of enrichment because feather condition scoring is an indirect measure of feather pecking behavior.

#### Footpad condition

Consistent with results of Da Costa et al. [46] and Krautwald-Junghanns et al. [47], our results indicated that footpad dermatitis worsened with age. Worsening footpad condition was observed as early as 16 wk for turkeys with pecking blocks, which was consistent with results reported by Krautwald-Junghanns et al. [47]. However, turkeys in the straw bale group showed no difference in footpad condition across all ages, and turkeys in the remaining treatment groups did not show the worst footpad condition until 19 wk.

***Gait*.** Several studies have documented a decrease in walking ability (worsening gait) as turkeys get older and heavier (e.g.[46, 48–50]), and our results are consistent with these reports as we observed a decline in gait with age in all treatment groups. Gait scores worsened in T and C turkeys from 16 wk onward and in turkeys in all other treatment groups at 19 wk; however, gait was not assessed between 16 and 19 wk. These results are similar to those of Da Costa et al. [46] who reported that gait was significantly worse in turkeys at 16–20 wk than at 13–15 wk, whereas Dalton et al. [48] documented a decline in gait from 13 wk onward. Stevenson et al. [49] also reported poorer gait when tom turkeys aged, as turkeys spent more time on both feet and took fewer steps per minute from 16 wk onward. Turkey walking ability can be affected by multiple environmental and management factors, such as environmental temperature and humidity level [12], and the specific genetic strain of turkey used. Further research will be beneficial in examining how various environmental and genetic factors influence age-related changes in turkey walking ability.

### Study limitations

As our study was conducted on a research farm, there are several limitations that need to be considered when interpreting the results. The group size and stocking density of the birds in each pen do not represent typical conditions on commercial turkey farms. Commercial turkey management guidelines for the turkeys used in this study suggest a stocking density of 2.5–3 birds/m^2^ [23]. However, the stocking density of the birds in this study (2.15 to 2.42 birds/m^2^) was lower than the commercial stocking densities to ensure enough space for the various enrichments. High stocking density and large group size are influential factors in promoting injurious pecking in turkeys, as well as other injuries such as broken wings due to hitting the walls, equipment or other birds because of insufficient space [51]. Other limiting factors in the present study were the size of the pen and the fixed locations of the feeders and the drinkers, which affected the placement of platforms (i.e. platforms could only be placed in one corner of the pen), which potentially limited turkey access and movement (two ramps were attached to platforms to ensure access). In addition, this study was conducted during the spring and summer seasons, which may not reflect possible seasonal factors that could influence injurious pecking behavior or walking ability. Future research will be valuable in examining variations among flocks and seasonal factors.

## Conclusion

Age affected the majority of welfare measures and gait in tom turkeys. Different types of environmental enrichment influenced feather quality and feather cleanliness but had little overall effect on gait under the conditions of this study. Providing tom turkeys with a straw bale and tunnel as environmental enrichment can help improve wing feather quality with age and providing straw bales may reduce the development of footpad dermatitis under suboptimal litter conditions. Providing enrichments that can help increase turkey activity and locomotion, including enrichment that can satisfy turkeys’ pecking and foraging needs (straw bale or pecking block) and elevated structures (bale or platform), may be beneficial for turkey walking ability, but further research is needed to investigate the links among activity level, space, enrichment, and walking ability.
